# Healthcare cost coverage inequality and its impact on hypertension and diabetes: A five‐year follow‐up study in a Malaysian rural community

**DOI:** 10.1002/hsr2.1880

**Published:** 2024-02-15

**Authors:** Adeola Folayan, Quek Kia Fatt, Mark Wing Loong Cheong, Tin Tin Su

**Affiliations:** ^1^ South East Asia Community Observatory (SEACO), Jeffrey Cheah School of Medicine and Health Sciences Monash University Malaysia Jalan Lagoon Selatan Bandar Sunway Selangor Malaysia; ^2^ Global Public Health, Jeffrey Cheah School of Medicine and Health Sciences Monash University Malaysia Jalan Lagoon Selatan Bandar Sunway Selangor Malaysia; ^3^ Department of Pharmacy Practice, School of Pharmacy Monash University Malaysia Jalan Lagoon Selatan Bandar Sunway Selangor Malaysia

**Keywords:** healthcare cost coverage, hypertension and diabetes, inequality, private health insurance

## Abstract

**Background and Aims:**

Inequality in health care access is a socioeconomic driver for non‐communicable disease related risk factors. This study examined the inequality trend in healthcare cost coverage (HCC) compared to private health insurance (PHI) coverage, a subtype of HCC, over 5 years. The study will also determine the association between HCC (and PHI) and the status of hypertension and diabetes diagnosis.

**Method:**

The rich‐poor ratio, concentration curve and concentration index were derived to determine the level of inequality. Furthermore, logistic regression was done to determine the association between HCC and the status of hypertension and diabetes.

**Results:**

The PHI group (rich‐poor ratio: 1.4 [rich: 454, poor: 314] and 2.6 [rich: 375, poor: 142]; concentration index: 0.123 [95% confidence interval, CI: 0.093–0.153] and 0.144 [95% CI: 0.109–0.178] in 2013 and 2018, respectively) has relatively higher inequality compared with the HCC group (rich‐poor ratio: 0.9 [rich: 307, poor: 337] and 1.1 [rich: 511, poor: 475]; concentration index: −0.027 [95% CI: −0.053 to −0.000] and −0.014 [95% CI: −0.033 to 0.006] in 2013 and 2018, receptively). Contrasting to the observation with the HCC group, PHI was associated with higher odds for hypertension (adjusted odds ratio [aOR] = 1.252, *p* = 0.01, 95% CI: 1.051–1.493) and diabetes (aOR = 1.287, *p* = 0.02, 95% CI: 1.041–1.590) in 2018.

**Conclusion:**

Over 5 years, the inequality in PHI coverage remained higher compared with HCC, which suggests that the rich enjoyed private healthcare more. Furthermore, those with PHI were more likely to report known hypertension and diabetes in 2018. It is reasonable to assume that those with PHI are more likely to have earlier diagnoses compared to others and are more likely to be aware of their condition. Policymakers need to identify strategies that can narrow the existing gap in quality and type of service between the private and public health sectors.

## Highlights

- There was inequality in PHI coverage over 5 years, suggesting that the rich enjoyed private healthcare more.
- Those with PHI were more likely to report known hypertension and diabetes in 2018. It is reasonable to assume that those with PHI are more likely to have earlier diagnoses compared to others and are more likely to be aware of their condition.
- The findings in this study could reinforce the need for policymakers to identify possible strategies to narrow the existing gap in quality and type of service between the private and public health sectors.

## 1 INTRODUCTION

The universal health coverage targets accessibility to health for all without the risk of financial impoverishment.^1^ Access to health includes physical access, financial security and acceptability. Financial security enables access to healthcare without financial barriers, mainly through healthcare cost coverage (HCC) plans that wholly or partly cater for healthcare expenditures.^2^, ^3^ These include health insurance plans, healthcare maintenance plans or other security plans that cover healthcare charges,^4^, ^5^ such as the guarantee letters for free healthcare services for eligible government workers in Malaysia.^6^

Health insurance plans could be social, community‐based or private health insurance (PHI).^5^ The PHI (a subtype of HCC) has provided a substantial platform to meet basic health financial demands with its wide coverage.^7^ However, PHI might not be affordable for low‐income people.^8^ The PHI often provides more quality service than other HCC plans.^9^ Hence, Malaysians preferably seek PHI if they can afford it, despite the affordable healthcare cost and the availability of other healthcare plans,^10^ giving the possibility of inequality in PHI uptake and distribution relative to other HCC plans. However, the PHI uptake in Malaysia is somewhat low.^9^ As of 2019, only 22% of the Malaysian population is estimated to have personal PHI.^11^ Being 50 years or older, female, Malay, a rural resident and having a lower education level are some factors that contribute to not having PHI in Malaysia.^9^

Inequality and inequity are both subjects of public health concerns. Inequality in access to healthcare services is a known health system policy challenge in low and middle‐income countries.^12^ Similarly, there is considerable literature evidence of the existence of inequity in the access to some healthcare services in many countries.^13^ Health inequality is the observable difference in health variables among different population subgroups.^14^ On the other hand, inequity is the unnecessary, avoidable, unfair and unjust systematic difference in the health status of different subgroups of a population.^15^, ^16^, ^17^ The term inequity is also used to describe inequality that still exists after adjusting the variable of fairness.^13^ Inequality highlights uneven distribution, while inequity highlights injustices or unfairness in distribution.^18^ Inequity analysis is a step further from inequality analysis as it incorporates fairness factors in distribution. Compared with inequality, inequity is widely seen as a better index to assess the distribution of healthcare variables. However, the concept of inequality, rather than inequity, was used in this study, as this study aimed to understand the dissimilarities in HCC distribution and not to explore the issue of injustices in HCC distribution. The issue of injustices will be addressed in further studies. Health inequality evaluation is important to understand where reforms are required to improve health equity.^14^ The common methods used to evaluate health inequality are the rich‐poor ratio, concentration curve, concentration index, Gini coefficient and slope index analysis.^6^, ^19^, ^20^, ^21^

Although HCC is a mechanism adopted to address healthcare service inequalities,^22^ HCC does not guarantee access to quality healthcare. Those with HCC could still report financial difficulties in access to desired healthcare, depending on their benefit package.^23^ Hence, disparities in HCC could lead to inequality in healthcare access and increasing trends of health inequalities, which invariably decrease some people's opportunities for better healthcare.

The prevalence of hypertension is increasing globally and has become an important risk factor for morbidity and mortality worldwide. As of 2015, more than 1 billion people globally are reported to have elevated blood pressure.^24^ Diabetes is also a common chronic non‐communicable disease (NCD) worldwide, with growing incidence and prevalence.^25^ As of 2014, 8.5% of the global adult population lives with diabetes worldwide.^26^ The Institute for Public Health in Malaysia reported that the prevalence of known diabetes increased from 8.3% to 9.4%, while the prevalence of known hypertension increased from 13.1% to 15.9% between 2015 and 2019.^10^, ^11^ The increasing prevalence of known hypertension and diabetes in Malaysia is expected to increase healthcare utilization for hypertension and diabetes management and prevent complications. Hence, a fair HCC distribution is important to ensure everyone gets prompt access to needed hypertension and diabetes care.

Limited access to healthcare facilities is a potential factor that could impede the appropriate management of NCDs.^22^ With increased healthcare utilization, health insurance is said to improve the health status of those insured.^27^ A study by Kressin et al.^28^ claimed that stable health insurance coverage was associated with good hypertension control. Brown et al.^29^ also reported improved diabetic outcomes with health insurance coverage. Hence, HCC possibly has a role to play in the diagnosis and management of hypertension and diabetes.

This study aims to assess the 5‐year trend in inequality of PHI distribution compared with HCC using data from the South‐East Asia Community Observatory (SEACO). It will also determine the association between HCC and the commonest NCDs (hypertension and diabetes).

## 2 METHODS

### 2.1 Data source and study design

This longitudinal study uses data from the SEACO, a certified health and demographic surveillance system.^26^ The data is part of community health surveys (2013 and 2018) conducted by SEACO in Segamat (a semi‐rural community in Johor, Malaysia). The dataset used for this analysis includes demographic data, income data, data on HCC and the status of hypertension and diabetes.

### 2.2 Ethical consideration

Participants provided written informed consent, and the study was approved by the Monash Human Research Ethics Committee: MUHREC 3837 for the health survey 2013 and MUHREC 13242 for the health survey 2018.

### 2.3 Inclusion and exclusion criteria

**Figure 1:**
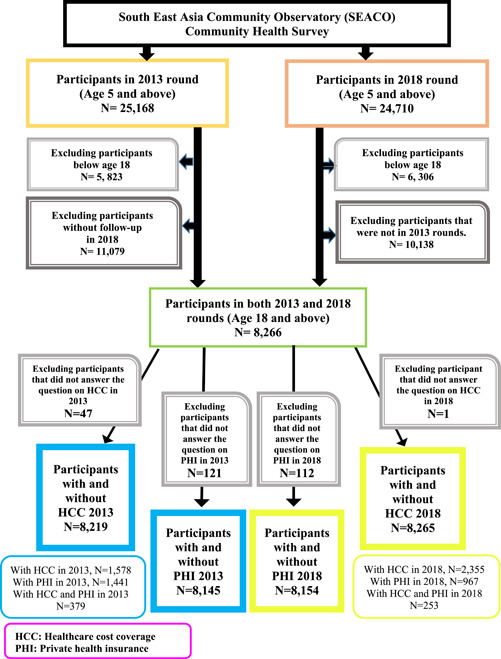
Flow chart of the study.

Everyone who participated in the 2013 and 2018 health rounds was eligible to be part of the study. Participants under 18 years and those who were lost to follow‐up in the 2018 survey were excluded from the study. The inclusion and exclusion flow chart is in Figure 1.

### 2.4 Variables

The dependent variables are known hypertension and diabetes. Known hypertension and diabetes were classified as yes if present and no if absent. The classification was based on previous medical diagnoses.

The independent variables are HCC and PHI. Participants are classified as having HCC if they have government or pensioner payment plans, employer‐provided health insurance, personal health insurance, employer or panel clinic payment plans or any other plan that provides HCC. Those who make out‐of‐pocket payments by themselves, family or household members are considered not to have HCC. Participants were asked if they have personal or employer‐provided PHI coverage in a separate question. Respondents who reported to have personal or employer‐provided PHI were considered to have PHI. Those who reported none were considered not to have PHI. One was coded for having HCC or PHI and zero for not having HCC or PHI. The demographic variables included are age, gender, ethnicity, marital status, educational level, employment status and household size.

### 2.5 Analysis

Five sets of analyzes were done. The first was the descriptive analysis, which includes the demographic distribution and the prevalence of HCC, PHI, hypertension and diabetes. The second was the rich‐poor ratio. The participants were ranked by household income from the poorest participant (as number one) to the richest participant (as number n, where n is the total number of participants). The household income is the combined average monthly income of all household members. The participants were then grouped into five: Q1, Q2, Q3, Q4, and Q5. Each group had 20% of the population. Q1 was the poorest group, while Q5 was the richest. The rich‐poor ratio was obtained by dividing the percentage of HCC (or PHI coverage) among individuals in Q5 by the percentage of HCC (or PHI coverage) among individuals in Q1. If the ratio is greater than one, the poor are disadvantaged; if the ratio is less than one, the poor are advantaged.^30^ Microsoft Excel was used for data organization and analysis. Although the rich‐poor ratio is easy to understand, it does not consider the data from other income groups between the richest and the poorest.^19^

The concentration curve, which was the third analysis, is also based on the income groups. It takes into account all data from all income groups (Q1, Q2, Q3, Q4, and Q5). It is a visual representation of the distribution of the health variable of interest.^20^ The concentration curve has a 45°C diagonal line, representing the equality line. If the contraction curve falls above the line, then the variable of interest is concentrated among the poorest. Similarly, if the contraction curve falls below the diagonal line, the variable of interest is concentrated among the richest. The cumulative percent of the health variable of interest in each income group, HCC and PHI, in this case, is plotted on the y‐axis, while the cumulative percent of the population (after being ranked from the poorest to the richest) is plotted on the x‐axis. Microsoft Excel was used for data organization and analysis.

The fourth analysis is the concentration index. One limitation of the concentration curve is that it does not have an index for comparing the magnitude of inequality^19^; hence, it is not very helpful in comparing different populations. This makes the concentration index more valuable compared with the concentration curve. The concentration index, Gini coefficient and slope index are common health inequality indexes. We have chosen the concentration index, the most used index for assessing health inequality relating to income status.^6^, ^21^ The concentration index takes a value between “+1” to “−1”. A negative value indicates that the variable of interest is concentrated among the poor, while a positive value indicates that the variable of interest is concentrated among the rich. The concentration index was estimated based on the household income using Stata SE 17. Suppose the health inequality is in favor of the rich; the concentration index could be multiplied by 75 to give the percentage of the health variable of interest to be redistributed to the poorer half from the richer half to give a distribution with the xero index.^31^

The last analysis was the multiple logistic regression analysis to determine if the coverage type (HCC and PHI) were predictive of the outcomes (known hypertension and diabetes). There was a separate model for each outcome (known hypertension and diabetes) in 2013 and 2018. The model was adjusted for socio‐demographic data such as age, gender, ethnicity, marital status, education, employment status, household income and household size. The analysis was done for participants aged 35 and above because SEACO only collects data on hypertension and diabetes for participants aged 35 and above. IBM SPSS Statistics, software version 27, was used for the rest of the data analysis. The adjusted odds ratio (aOR) and 95% confidence interval (CI) were used to report the size of the association. A *p* < 0.05 was considered statistically significant. All statistical analyzes were two‐sided.

## 3 RESULT

The socio‐demography distribution of participants, the percentages of participants with HCC or PHI and the prevalence of hypertension and diabetes are shown in Table 1. The socio‐demography distribution followed a similar pattern for both groups in 2013 and 2018. The percentage of participants with HCC increased from 2013 to 2018 (2013: 19.2% [1578 of 8219], 2018: 28.5% [2355 of 8265]), while the percentage of participants with PHI decreased (2013: 17.7% [1441 of 8145], 2018: 11.9% [967 of 8154]). The prevalence of hypertension and diabetes increased for both groups from 2013 to 2018 (HCC: hypertension, 23.3% [1621 of 6967] to 37.3% [2779 of 7453], diabetes, 12.6% [878 of 6967] to 21.1% [1576 of 7453]; PHI: hypertension, 23.2% [1601 of 6908] to 37.3% [2748 of 7359], diabetes, 12.6% [870 of 6908] to 21.1% [1555 of 7359]).

**Table 1:** Characteristics of participants in the study.

Variables	With/without healthcare cost coverage in 2013 (*n* = 8219)	With/without healthcare cost coverage in 2018 (*n* = 8265) *n* (%)	With/without private health insurance in 2013 (*n* = 8145)	With/without private health insurance in 2018 (*n* = 8154)
	*n* (%)	*n* (%)	*n* (%)	*n* (%)
**Age**				
18–34	1252 (15.2)	1261 (15.3)	1237 (15.2)	1233 (15.1)
35–44	1231 (15.0)	1237 (15.0)	1221 (15.0)	1218 (14.9)
45–54	1917 (23.3)	1934 (23.4)	1898 (23.3)	1907 (23.4)
55–64	2352 (28.3)	2360 (28.6)	2333 (28.3)	2335 (28.6)
≥65	1467 (17.8)	1473 (17.8)	1456 (17.9)	1461 (17.9)
**Gender**				
Female	4946 (60.2)	4982 (60.3)	4921 (60.4)	4925 (60.4)
Male	3273 (39.8)	3283 (39.7)	3224 (39.6)	3229 (39.6)
**Ethnicity**				
Malay	5419 (65.9)	5446 (65.9)	5360 (65.8)	5370 (65.9)
Chinese	1695 (20.6)	1709 (20.7)	1687 (20.7)	1683 (20.6)
Indian	942 (11.5)	946 (11.4)	934 (11.5)	938 (11.5)
Others	162 (2.0)	163 (2.0)	163 (2.0)	162 (2.0)
*Missing*	1 (0.0)	1 (0.0)	1 (0.0)	1 (0.0)
**Marital status**				
Married	6476 (78.8)	6508 (78.7)	6416 (78.8)	6424 (78.8)
Never married	743 (9.0)	750 (9.1)	739 (9.1)	735 (9.0)
Divorced	103 (1.3)	103 (1.2)	102 (1.3)	101 (1.2)
Widow(er)	786 (9.6)	793 (9.6)	778 (9.6)	786 (9.6)
Others	88 (1.1)	88 (1.1)	87 (1.1)	86 (1.1)
*Missing*	23 (0.3)	23 (0.3)	23 (0.3)	22 (0.3)
**Education**				
No formal education	224 (2.7)	226 (2.7)	223 (2.7)	223 (2.7)
Primary	6229 (75.8)	6259 (75.7)	6172 (75.8)	6174 (75.7)
Secondary	168 (2.0)	168 (2.0)	161 (2.0)	161 (2.0)
Tertiary	263 (3.2)	264 (3.2)	255 (3.1)	258 (3.2)
Others	1234 (15.0)	1242 (15.0)	1228 (15.1)	1233 (15.1)
*Missing*	101 (1.2)	106 (1.3)	106 (1.3)	105 (1.3)
**Employment status**				
Paid employee	2152 (26.2)	2160 (26.2)	2119 (26.0)	2119 (26.0)
Self‐employed	1467 (17.8)	1472 (17.8)	1445 (17.7)	1453 (17.8)
Housewife/Husband	3040 (27.0)	3058 (37.0)	3026 (37.2)	3030 (37.2)
Not working	1043 (12.7)	1054 (12.8)	1046 (12.8)	1044 (12.8)
Pensioner	498 (6.1)	498 (6.0)	489 (6.0)	489 (6.0)
*Missing*	19 (0.2)	20 (0.2)	20 (0.2)	19 (0.2)
**Household income (RM)**				
B40 (≤4850)	7791 (94.8)	7837 (94.8)	7732 (94.9)	7730 (94.8)
M40 (4851–10,970)	385 (4.7)	385 (4.7)	372 (4.6)	382 (4.7)
T20 (≥10,971)	40 (0.5)	40 (0.5)	38 (0.5)	39 (0.5)
*Missing*	3 (0.0)	3 (0.0)	3 (0.0)	3 (0.0)
**Household size**				
<4	3480 (42.3)	3498 (42.3)	3459 (42.5)	3463 (42.5)
=4	1355 (16.5)	1365 (16.5)	1355 (16.6)	1336 (16.4)
>4	3352 (40.8)	3370 (40.8)	3331 (40.9)	3324 (40.8)
*Missing*	32 (0.4)	32 (0.4)	0 (0.0)	31 (0.4)
**With coverage**				
Yes	1578 (19.2)	2355 (28.5)	1441 (17.1)	967 (11.9)
No	6641 (80.8)	5910 (71.5)	6704 (82.3)	7187 (88.1)
**≥35 years only**	**(*n*** = **6967)**	**(*n*** = **7453)**	**(*n*** = **6908)**	**(*n*** = **7359)**
**Hypertension (%)**				
Yes	1621 (23.3)	2779 (37.3)	1601 (23.2)	2748 (37.3)
No	5318 (76.3)	4671 (62.7)	5280 (76.4)	4608 (62.6)
*Missing*	28 (0.5)	3 (0.0)	27 (0.4)	3 (0)
**Diabetes (%)**				
Yes	878 (12.6)	1576 (21.1)	870 (12.6)	1555 (21.1)
No	6055 (86.9)	5875 (78.8)	6005 (86.9)	5802 (78.8)
*Missing*	34 (0.5)	2 (0.0)	33 (0.5)	2 (0.0)

*Note*: USD 1 = RM 3.086 (June 01, 2013) and RM 3.979 (June 01, 2018).

**Figure 2:**
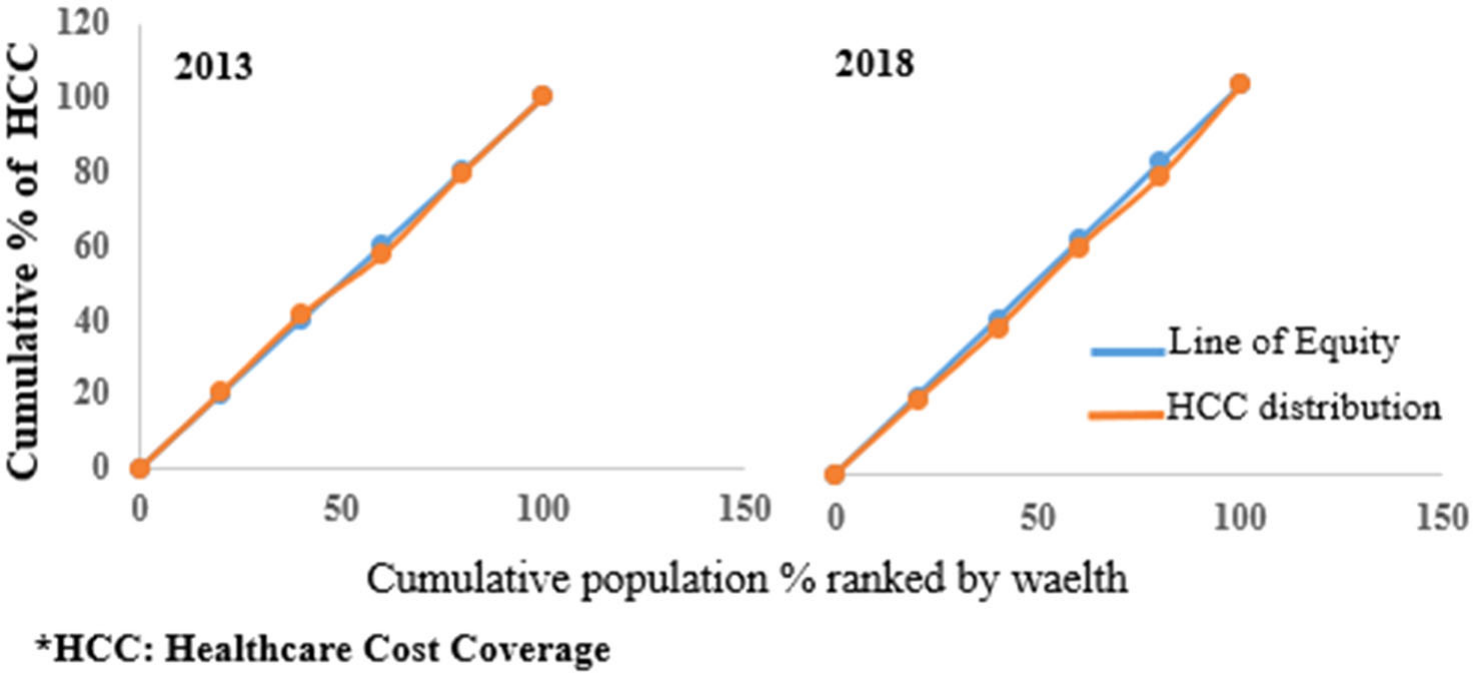
Concentration curve for healthcare cost coverage.

**Figure 3:**
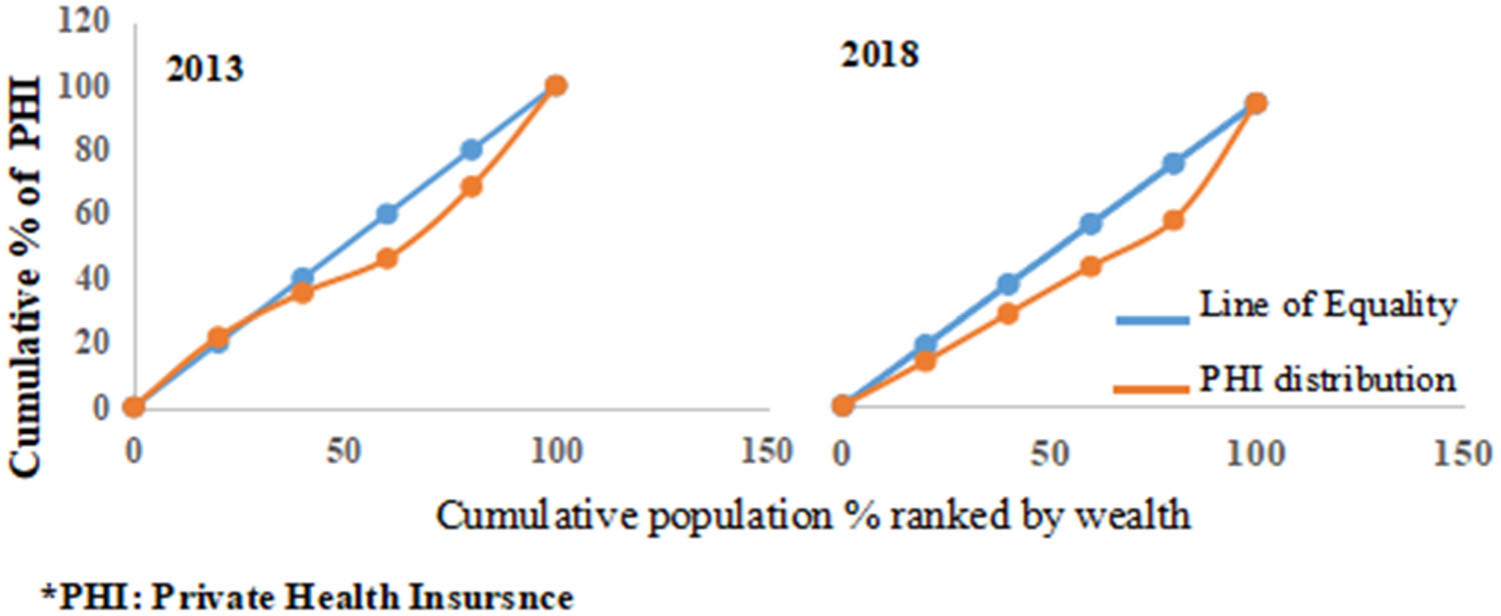
Concentration curve for private health insurance.

The concentration curves for the HCC and PHI are shown in Figures 2 and 3, respectively. As indicated in Figure 2, the concentration curves were very close to the line of equality in 2013 and 2018. This suggests no visible inequality in the distribution of HCC. The situation is slightly different for the PHI distribution. The PHI concentration curve in Figure 3 falls below the line of equality in 2013 and 2018, indicating that PHI is more concentrated among those with higher income rankings. As shown in Table 2, the rich‐poor ratio was highest for the PHI in 2018, showing a rising trend in the rich‐poor ratio from 1.4 (rich: 454, poor: 314) to 2.6 (rich: 375, poor: 142) over 5 years. This is similar to the distribution of HCC. The rich‐poor ratio increased for HCC from 0.9 (rich: 307, poor: 337) to 1.1 (rich: 511, poor: 475) from 2013 to 2018. The concentration index (Table 2) for PHI had positive values (concentration index: 0.123 [95% CI: 0.093–0.153] and 0.144 [95% CI: 0.109–0.178] in 2013 and 2018, respectively), indicating inequality in favor of the rich. This is to say that PHI was concentrated among the rich in 2013 and 2018. The concentration index was multiplied by 75 to obtain the percentage of coverage that must be redistributed to the poor to attain equality in PHI distribution. The percent to be redistributed was highest for PHI in 2018 at 10.8% (concentration index (0.144) × 75).

**Table 2:** Rich‐poor ratio and concentration index for HCC and PHI.

Year	Healthcare cost coverage				Private health insurance			
	Rich‐poor ratio	CIX	95% CI	CIX × 75 (%)	Rich‐poor ratio	CIX	95% CI	CIX × 75 (%)
2013	0.9 (rich: 307, poor: 337)	−0.027	−0.053 to −0.000	2.2	1.4 (rich: 454, poor: 314)	0.123	0.093 to 0.153	9.2
2018	1.1 (rich: 511, poor: 475)	−0.014	−0.033 to 0.006	1.1	2.6 (rich: 375, poor: 142)	0.144	0.109 to 0.178	10.8

Abbreviations: CI, confidence Interval; CIX, concentration index; CIX × 75, CIX multiplied by 75 to obtain the percentage of coverage to be redistributed to attain equality; HICC, healthcare cost coverage; PHI, private health insurance.

Table 3 shows that HCC was associated with the prevalence of hypertension (2013: aOR = 0.638, *p* < 0.001, 95% CI: 0.553–0.737 and 2018: aOR = 0.637, *p* < 0.001, 95% CI: 0.572–0.711) and diabetes (2013: aOR = 0.620, *p* < 0.001, 95% CI: 0.521–0.737 and 2018: aOR = 0.699, *p* < 0.001, 95% CI: 0.617–0.791) in 2013 and 2018. These show that those with HCC are less likely to report known hypertension and diabetes. PHI in 2018 was also associated with hypertension (aOR = 1.252, *p* = 0.01, 95% CI: 1.051–1.493) and diabetes (aOR = 1.287, *p* = 0.02, 95% CI: 1.041–1.590), but no association was recorded in 2013. Hence, those with PHI were more likely to report hypertension and diabetes in 2018 but not in 2013.

**Table 3:** Multiple logistic regression of healthcare cost coverage and private health insurance on the prevalence of hypertension and diabetes (only for participants aged 35 and above).

Insurance type/year	Hypertension				Diabetes			
	*n*	aOR	*p* value	95% CI	*n*	aOR	*p* value	95% CI
HCC 2013	6808	0.638	<0.001	0.553–0.737	6805	0.620	<0.001	0.521–0.737
HCC 2018	7442	0.637	<0.001	0.572–0.711	7443	0.699	<0.001	0.617–0.791
PHI 2013	6762	1.172	0.08	0.981–1.401	6759	1.123	0.32	0.894–1.411
PHI 2018	7348	1.252	0.01	1.051–1.493	7349	1.287	0.02	1.041–1.590

*Note*: Socio‐demographic data adjusted for were age, gender, ethnicity, marital status, education, employment status, household income and household size. Predictor: HCC and PHI, Reference group: without HCC or PHI. Code: 1 (with HCC or PHI) and 0 (without HCC or PHI). Outcome variables: Hypertension and Diabetes.

Abbreviations: aOR, adjusted odds ratio; CI, confidence interval; HCC, healthcare cost coverage; n, number of participants; PHI, private health insurance,

## 4 DISCUSSION

The prevalence of HCC increased while the prevalence of PHI decreased from 2013 to 2015. PHI had higher inequality in favor of the rich compared with HCC for all the inequality indicators measured. The prevalence of known hypertension and diabetes increased from 2013 to 2018 for both HCC and PHI groups. Those with HCC were less likely to report known hypertension and diabetes in 2013 and 2018. Those with PHI were more likely to report known hypertension and diabetes in 2018. PHI was not associated with known hypertension and diabetes in 2013.

The increase in the prevalence of HCC and the simultaneous decrease in the prevalence of PHI gives a clue to the higher inequality observed with PHI coverage. This suggests that some insurees might have migrated from PHI to other affordable HCC plans or lost their health insurance coverage. Hence, over the 5 years, there was a reduction in the number of people who could afford the PHI, contributing to the inequality in PHI distribution observed in this study. This is similar to the observation at the national level. The national health and morbidity survey report in Malaysia states that the prevalence of employer‐sponsored health insurance and PHI decreased from 10.4% and 22.3% in 2015 to 7.6% and 16.5% in 2019, respectively.^10^, ^11^ In 2019, 43% of people without private insurance claimed they did not have a private insurance plan because they could not afford it.^27^ This clearly shows that there is inequality in the distribution of PHI. This has been an issue of concern in Malaysia as certain income groups might not enjoy the desired healthcare. In response to this and other healthcare financing concerns, the Malaysian government implemented the *Skim Peduli Kesihatan* (PeKa B40) initiative for the B40 in 2019 to cater for certain screening and treatment needs. Other initiatives include the mySalam and the *Bantuan Sara Hidup* program, among others, to mitigate financial barriers to access to healthcare.^6^ This implies that those with HCC could encounter financial barriers to accessing certain healthcare, depending on their HCC type. Hence, inequality in access to healthcare might be minimal for HCC in general but higher for some HCC subtypes.

HCC was associated with known hypertension and diabetes in 2013 and 2018. Those with HCC have a lower likelihood of reporting known hypertension and diabetes. This is similar to the observation by Mpofu et al.^32^ in a cross‐sectional survey in Brazil. In their study, they claimed that women without PHI are more likely to report known hypertension (aRR = 1.4 95% CI: 1.1–1.7). Malta et al.^33^ also reported that the prevalence of self‐reported hypertension and diabetes in Brazil was less among the population with health insurance compared with those without health insurance. However, the lower likelihood of the participants with HCC reporting known hypertension and diabetes might be an indication that participants may not have efficient contact with the health system even though they have HCC. It highlights a need to reinforce NCD surveillance in community settings. The study by Chandran et al. in 2020 examined the NCD surveillance tools, activities and performance in Malaysia in line with the World Health Organization Global Monitoring Framework for NCDs. They highlighted that Malaysia has a robust NCD Surveillance system for some NCD indicators; nevertheless, some aspects of the NCD Surveillance system require strengthening.^34^

However, the observation was different for the group with PHI. In 2018, PHI was associated with the presence of known hypertension and diabetes. Those with PHI were more likely to report known hypertension and diabetes. This shows that those with PHI probably have more access to healthcare facilities, which might facilitate the diagnosis and management of known hypertension and diabetes, as healthcare access has been linked to both hypertension diagnosis and treatment.^35^ Hence, those with PHI are more likely to be aware of their condition. Another possibility is that the rich with high‐risk conditions may have opted for PHI for more efficient healthcare since they can afford it. This suggests a case of adverse selection for the richer group since known hypertension and diabetes were not associated with PHI at baseline. Adverse selection occurs when an individual purchases health insurance because he anticipates the need for health care due to their health risk.^36^ The poor with high‐risk conditions might not have this option due to inequality in PHI distribution. Although, adverse selection should not be of concern in developing countries where the health insurance system is still growing.^37^ However, where the country's health insurance system features both public (or basic) and private insurance, the public (or basic) insurance should cover treatments prone to adverse selection.^38^ This will simultaneously solve the problem of inequality and adverse selection for the insurance market.

There were a few limitations in this study. Known hypertension and diabetes were self‐reported; hence, the possibility of reporting errors cannot be ruled out. Second, the assessment of coverage type (HCC and PHI) and the prevalence of NCDs (hypertension and diabetes) was done simultaneously, making it impossible to establish a causal relationship. Another limitation is that this study used the inequality index rather than inequity. Inequity estimation is a more robust analysis in healthcare financing as it takes into account the ability to pay, which involves a sense of fairness. However, inequality stands as a base for further studies relating to inequity.

## 5 CONCLUSION

This study examines the inequality trend in HCC compared to PHI over 5 years. The higher inequality in PHI coverage, compared with HCC plans over 5 years, suggests that the rich might be enjoying better healthcare services. Furthermore, those with PHI were more likely to report known hypertension and diabetes in 2018. It is reasonable to assume that those with PHI are more likely to have earlier diagnoses compared to others and are more likely to be aware of their condition. The findings in this study could reinforce the need for policymakers to identify strategies that can narrow the existing gap in quality and type of service between the private and public health sectors or increase access to private health services for the lower‐income group that desires it.

## AUTHOR CONTRIBUTIONS

**Adeola Folayan**: formal analysis; investigation; methodology; validation; visualization; writing—original draft; writing—review & editing. **Quek Kia Fatt**: methodology; supervision; writing—review & editing. **Mark Wing Loong Cheong**: methodology; supervision; writing—review & editing. **Tin Tin Su**: conceptualization; methodology; supervision; writing—review & editing.

## CONFLICT OF INTEREST STATEMENT

The authors declare no conflicts of interest.

## TRANSPARENCY STATEMENT

The lead author Tin Tin Su affirms that this manuscript is an honest, accurate, and transparent account of the study being reported; that no important aspects of the study have been omitted; and that any discrepancies from the study as planned (and, if relevant, registered) have been explained.

## Data Availability

The dataset analyzed for the current study is not publicly available. Requests to access the datasets should be directed to the corresponding author.
